# Detection of *Bartonella tamiae, Coxiella burnetii* and rickettsiae in arthropods and tissues from wild and domestic animals in northeastern Algeria

**DOI:** 10.1186/s13071-016-1316-9

**Published:** 2016-01-20

**Authors:** Hamza Leulmi, Atef Aouadi, Idir Bitam, Amina Bessas, Ahmed Benakhla, Didier Raoult, Philippe Parola

**Affiliations:** Aix Marseille Université, Unité de Recherche en Maladies Infectieuses et Tropicales Emergentes (URMITE), UM63, CNRS 7278, IRD 198 (Dakar), Inserm 1095, Faculté de Médecine, 27 bd Jean Moulin, 13385 Marseille, Cedex 5 France; Ecole Nationale Supérieure Vétérinaire d’Alger. El Aliya Alger, Algiers, 16000 Algeria; Département des Sciences Vétérinaires, Université Cherif Messaadia, Souk Ahras, 41000 Algeria; Département des Sciences Vétérinaires, Université Chadli Bendjdid, El Tarf, 36000 Algeria; Laboratoire d’Ecologie et Environnement: Interaction, Génome, Université de Bab Ezzouar, Bab Ezzouar, 16000 Algeria

**Keywords:** *Bartonella tamiae*, *Rickettsia*, *Coxiella burnetii*, Ticks, Fleas, Algeria

## Abstract

**Background:**

In recent years, the scope and importance of emergent vector-borne diseases has increased dramatically. In Algeria, only limited information is currently available concerning the presence and prevalence of these zoonotic diseases. For this reason, we conducted a survey of hematophagous ectoparasites of domestic mammals and/or spleens of wild animals in El Tarf and Souk Ahras, Algeria.

**Methods:**

Using real-time PCR, standard PCR and sequencing, the presence of *Bartonella* spp., *Rickettsia* spp., *Borrelia* spp. and *Coxiella burnetii* was evaluated in 268/1626 ticks, 136 fleas, 11 *Nycteribiidae* flies and 16 spleens of domestic and/or wild animals from the El Tarf and Souk Ahras areas.

**Results:**

For the first time in Algeria, *Bartonella tamiae* was detected in 12/19 (63.2 %) *Ixodes vespertilionis* ticks, 8/11 (72.7 %) *Nycteribiidae* spp. flies and in 6/10 (60 %) bat spleens (*Chiroptera* spp.). DNA from *Coxiella burnetii*, the agent of Q fever, was also identified in 3/19 (15.8 %) *I. vespertilionis* from bats. *Rickettsia slovaca*, the agent of tick-borne lymphadenopathy, was detected in 1/1 (100 %) *Haemaphysalis punctata* and 2/3 (66.7 %) *Dermacentor marginatus* ticks collected from two boars (*Sus scrofa algira*) respectively. *Ri. massiliae*, an agent of spotted fever, was detected in 38/94 (40.4 %) *Rhipicephalus sanguineus* sensu lato collected from cattle, sheep, dogs, boars and jackals. DNA of *Ri. aeschlimannii* was detected in 6/20 (30 %) *Hyalomma anatolicum excavatum* and 6/20 (30 %) *Hy. scupense* from cattle. Finally, *Ri. felis*, an emerging rickettsial pathogen, was detected in 80/110 (72.7 %) *Archaeopsylla erinacei* and 2/2 (100 %) *Ctenocephalides felis* of hedgehogs (*Atelerix algirus*).

**Conclusion:**

In this study, we expanded knowledge about the repertoire of ticks and flea-borne bacteria present in ectoparasites and/or tissues of domestic and wild animals in Algeria.

## Background

Since the beginning of the 20th century, ticks (Acarina), fleas (Siphonaptera) and other hematophagous arthropods have been implicated as vectors, reservoirs, and/or amplifiers of agents of human zoonoses [1]. Ticks are hematophagous arthropods that are considered second vectors (after mosquitoes) of human disease and the most significant vectors of disease-causing pathogens in animals [2, 3]. Ticks can transmit a broad range of pathogens, including viruses, protozoa and bacteria [4]. Likewise, fleas are also able to transmit several agents of infectious diseases [5]. The transmission of these zoonotic agents to humans occurs mainly through their bites or inoculation of their infected feces into pruritic bite lesions [6–9]. Rickettsioses, bartonelloses and Q fever are vector-borne diseases that may be severe and that have a widespread geographical distribution.

*Rickettsia* spp., the etiological agent of rickettsioses, are intracellular Gram-negative bacteria that represent an emergent global threat [10]. *Ri. felis,* an emerging pathogen, and *Ri. typhi,* the agent of murine typhus (MT), are the main rickettsial pathogens associated with fleas [11], belonging to the spotted fever group (SFG) [12] and typhus group of rickettsiae, respectively [13]. Most of the SFG are transmitted by ticks [14] that are widely distributed in northern Africa [15, 16]. In Algeria, 11 rickettsial pathogens have been detected in ticks, fleas, lice and humans, including *Ri. conorii* subspecies *conorii*, *Ri. aeschlimannii*, *Ri. sibirica mongolitimonae*, *Ri. massiliae*, *Ri. slovaca*, *Ri. helvetica*, *Ri. africae, Ri. monacensis, Ri. felis, Ri. typhi* and *Ri. prowazekii* [17].

Likewise, bartonelloses are diseases caused by fastidious, hemotropic bacteria from the genus *Bartonella* [18] which parasitize erythrocytes or epithelial cells across a range of mammalian hosts, including humans, rodents and chiroptera [19–21]. In Algeria, few investigations into the diversity of *Bartonella* spp*.* from animals and vectors have been conducted. Namely, *B. vinsonii* subsp. *berkhoffii*, *B. clarridgeiae*, and *B. elizabethae* were detected infecting domestic dogs [22, 23] and fleas collected from hedgehogs [24], *B. henselae* was isolated from stray cats [25] and *B. rochalimae* was detected in fleas collected from brown rats (*Rattus norvegicus*) [24].

In addition, *Coxiella burnetii*, the causative agent of Q fever, is a highly infectious zoonotic intracellular bacterium which can affect different species of wild and domestic mammals; it can also infect arthropods and birds [26]. In Algeria few human cases of Q fever have been documented, with only two human cases reported in Oran [27].

The goal of this investigation was to assess the presence of emerging zoonotic bacteria in ectoparasites and tissues sampled from wild and domestic animals present in northeastern Algeria.

## Methods

### Study areas

The first part of the study was conducted in May 2012 in El Ghorra (Bougous, El Tarf) (36° 39′ 34″ N 8° 22′ 10″ E) in the far northeast of Algeria. El Ghorra is a humid bioclimatic zone. It was the highest site in the study area, where the maximum altitude is 1202 m (Jebel El Ghorra). The relief of El Ghorra is characterized by a set of wooded mountains forming the “forest of El Ghorra”. Its coverage includes 96 % vegetation and 2 % herbaceous layer, of which 500 ha are occupied by cork oak (*Quercus suber*) and 600 ha by canary oak, locally known as zeen oak (*Quercus canariensis*) [28].

The second part of this work was performed in July 2013, in Cheabat El Balout (Ouled Driss, Souk Ahras) in northeastern Algeria near El Tarf, (36° 22′ 01.30″ N 08° 07′ 27.48″ E). This study area is mountainous and is located at 1000 m above sea level, representing an extension of the Telli Atlas. It has a semi-humid climate characterized by hot summers and cold, wet winters with a rainfall averaging 800 mm per year.

### Ectoparasite collection and tissue sampling

The investigation in El Ghorra (Bougous, El Tarf) was conducted on domestic animals (cattle, sheep, goats and dogs). Ectoparasites were collected with the permission of the animals’ owners. All arthropods were collected using blunted watchmakers forceps and immediately placed in tubes of 70 % ethanol labeled with the identification number and the date of collection. A portion of the collected ectoparasites was used for the present study.

The field sampling in Cheabat El Balout (Ouled Driss, Souk Ahras) was conducted on wild mammals [two boars (*Sus scrofa algira*), two jackals (*Canis aureus*) one mongoose (*Echinomon herpestis*), ten bats (*Chiroptera* spp.), one porcupine (*Hystrix cristata*) and four hedgehogs (*Atelerix algirus*)]. Ectoparasites and tissues (spleens) were sampled. Hedgehogs were captured with the aid of flashlights during nightly walks through parts of the study regions near a poultry slaughterhouse. Hedgehogs were anesthetized using ketamine and released into their natural habitat after full recovery of ectoparasites.

Two boars, two jackals, one mongoose and one porcupine were found recently dead following road accidents and were also inspected for ectoparasites, and their spleens were sampled using adapted scalpels and stored in tubes containing 70 % ethanol. Finally, we caught ten bats using hunting nets. The nets, identical to those used by ornithologists to capture and band birds, are very fine, and similar to a mesh-like fishing net. They are stretched between two poles, and placed at the entrance of the bat cave. Once detached from the nets, we searched for ectoparasites and took spleen samples.

All biological materials were forwarded thereafter to Marseille, France for morphological identification of ectoparasites at the species level using morphological criteria within standard taxonomic keys [29, 30]. Molecular analyses of ectoparasites and tissue samples were performed to detect *Rickettsia* spp., *Bartonella* spp.*, Coxiella burnetii* and *Borrelia* spp.

### Ethical approval

The study on hedgehogs was authorized by the local ethics committee and by national legislation (le journal officiel n° 47 du 19 juillet 2006, http://www.iucnredlist.org/apps/redlist/details/27926/0). Oral permission to place nets to trap bats in the study area was granted by the landowner, who placed his pets inside the cave in the rainy days. At the beginning of the study, the work on bats was programmed only on bats ectoparasites and when bats were recovered the next day, they were dead, that because we proceeded bats spleen also. In addition Algeria does not have ethical committee of bats. All experiments were done under the supervision of the Ministry of Health of Algeria.

### DNA extraction

Prior to DNA extraction, a convenient sample was selected according to a good representation of species and hosts in El Ghorra (samples < 20 were all processed while for samples > 20 only 20 samples were processed). All collected ectoparasites and biological materials from Cheabat El Balout were used to extract their DNA.

Arthropods and spleens were rinsed twice in distilled water for 10 min and dried on sterile filter paper; handling was performed in a laminar flow biosafety cabinet. Ectoparasites and a portion of the spleen samples were individually crushed in sterile Eppendorf tubes. Total DNA was extracted in a final volume of 200 μl from one half of each ectoparasite and a portion of the spleen using the QIAamp Tissue Kit (Qiagen, Hilden, Germany) by Qiagen-BioRobot EZ1, according to the manufacturer’s instructions. Genomic DNA was stored at −22 °C under sterile conditions.

### Detection of bacteria

Once DNA had been extracted, it was used in qPCR template assays to detect *Bartonella* spp., *Rickettsia* spp., *Coxiella burnetii* and *Borrelia* spp. The final qPCR reaction mixture consisted of 5 μl of DNA and 15 μl of mix from the Takyon PCR Kit (Qiagen, Hilden, Germany) as described [31]. Negative controls were used in each qPCR and consisted of DNA extracted from uninfected ticks from our laboratory colony. Positive controls included DNA extracted from a dilution of cultured strains of *B. elizabethae* (detection of *Bartonella* spp.), *Ri. montanensis* (for the detection of *Rickettsia* spp.), *Coxiella burnetii* (for the detection of *Coxiella burnetii*) and *Borrelia crocidurae* (for the detection of *Borrelia* spp.). Results were deemed positive if the Cycle threshold (Ct) value obtained by CFX96 was lower than 36. All positive results were confirmed with a second qPCR system and/or sequence reaction.

### Detection of *Bartonella* spp.

DNA samples were screened by qPCR targeting the ITS for the detection of *Bartonella* spp. [32]. The positive samples with ITS primers were then confirmed by standard PCR performed with *Bartonella-*specific primers of the intergenic spacer region between the 16S and 23S rRNA genes [33]. PCR amplification success was verified by migration in 2 % Agarose gel, followed by purification using the NucleoFast 96 PCR plate (Machery-Nagel EURL, France), as recommended by the manufacturer. The purified PCR products were sequenced using Urb1 and Urb2 primers and using BigDye version 1.1 Cycle Sequencing Ready Reaction Mix (Applied Biosystems, Foster City, CA). Data were collected with an ABI Prism 3130xl Genetic Analyzer capillary sequencer (ABI PRISM, PE Applied Biosystems, USA). Sequences were edited and assembled using Chromas Pro 1.34 (Technelysium Pty. Ltd., Tewantin, Australia). BLAST searches were performed to identify the obtained sequences.

### Detection of *Rickettsia* spp.

Rickettsial DNA was detected using a Rickettsia genus-specific qPCR with a 25-bp probe targeting the partial sequence of the citrate synthase gene (*gltA*) [34]. All tick samples identified as positive by qPCR were confirmed by a different standard PCR and sequencing for the fragments of *OmpA* gene [34]. DNA sequencing reactions were performed on highly positive samples (Ct <28). Tick samples with Ct > 28 were screened by qPCR specific to the species according to the sequencing result using *Ri. slovaca*, *Ri. massiliae* or *Ri. aeschlimannii* qPCRs systems (Table 1), while flea samples testing positive with the RKND03 qPCR were directly tested with two qPCRs systems targeting the biotin synthase (*bioB*) and membrane phosphatase genes of *Ri. felis* [35]. Table 1 summarizes the probes and primers used to confirm and identify rickettsiae in samples.

**Table 1:** Target sequences, primers and probes used to confirm the detection of rickettsiae by qPCR

Quantitative real-time PCR designation and specificity	qPCR Sytem used	Forward primer	Reverse primer	Probe
*Rickettsia felis*	Biotin synthase	ATG-TTC-GGG-CTTCCG-GTA-TG	CCG-ATT-CAG-CAGGTT-CTT-CAA	6-FAM- GCT-GCG-GCGGTA-TTT-TAG-GAA -TGGG-TAMRA
*Rickettsia aeschlimannii*	Scal	AAGCGGCACTTTAGGTAAAGAAA	CATGCTCTGCAAATGAACCA	6FAM-TGGGGAAATATGCCGTATACGCAAGC -TAMRA
*Rickettsia massiliae*	R.mass_9666	CCAACCTTTTGTTGTTGCAC	TTGGATCAGTGTGACGGACT	6FAM-CACGTGCTGCTTATACCAGCAAACA -TAMRA
*Rickettsia slovaca*	R.slov	GCAACGGTTTTTGGTATCGT	AATCGAATGCACCACCACTT	6FAM- TCCCGTCCCAGCCATTCGTC -TAMRA

### Detection of *Borrelia* spp.

qPCR targeting the 16S rRNA gene was used, as described elsewhere [34], to screen DNA samples for all *Borrelia* spp.

### Detection of *Coxiella burnetii*

*Coxiella burnetii* bacterial DNA was initially detected by qPCR with *C. burnetii*–specific primers and a probe designed to amplify the IS1111 gene [36]. qPCR with primers and a probe designed for the amplification of IS30a spacers were used to confirm *C. burnetii*–positive results [27].

## Results

### Sample collection and ectoparasites identification

In El Ghorra, a total of 1549 ticks (Table 2) were collected, including eight species; 565 ticks were sampled from 123 cattle, 529 ticks from 250 sheep, 130 ticks were collected from 125 goats and 325 ticks were sampled from 50 dogs.

**Table 2:** Detection of Rickettsiae, *Bartonella* spp., and *Coxiella burnetii* in arthropods

Ectoparasite species	Localization	Animal (N)	No. of ectoparasites collected (m = male, f = female)	No. of ectoparasites tested by qPCR (m = male, f = female)	*Rickettsia spp*	*Bartonella spp*	*Coxiella burnetii*
*Rhipicephalus sanguineus*	El Ghorra, El Tarf.	Cattle (123)	316 (104 m, 212f)	20 (10 m, 10f)	3/20 (1 m, 2f) *Ri. massiliae*	-	-
		Sheep (250)	454 (217 m, 237f)	20 (10 m, 10f)	11/20 (9 m, 2f) *Ri. massiliae*	-	-
		Goats (128)	104 (55 m, 49f)	20 (10 m, 10f)	-	-	-
		Dogs (50)	323 (222 m, 101f)	20 (10 m, 10f)	1/20 (1 m) *Ri. massiliae*	-	-
	Cheabat El Balout, Souk Ahras	Boars (2)	9 (5 m, 4f)	9 (5 m, 4f)	8/9 (5 m, 3f) *Ri. massiliae*	-	-
		Mangoose (1)	2 (1 m, 1f)	2 (1 m, 1f)	2/2 (1 m, 1f) *Ri. massiliae*	-	-
		Jackals (2)	23 (15 m, 8f)	23(15 m, 8f)	13/23(8 m, 5f) *Ri. massiliae*	-	-
		Hedgehogs (4)	10 (2 m, 8f)	10(2 m, 8f)	-	-	-
*Rhipicephalus bursa*	El Ghorra, El Tarf.	Cattle (123)	50 (39 m, 11f)	20 (10 m, 10f)	2/20 (2f) *Ri. massiliae*	-	-
		Sheep (250)	72 (37 m, 35f)	20 (10 m, 10f)	-	-	-
		Goats (128)	19 (19 m)	19 (19 m)	-	-	-
*Hyalomma lusitanicum*	El Ghorra, El Tarf.	Sheep (250)	1 (1 m)	1 (1 m)	-	-	-
		Goats (128)	3 (2 m, 1f)	3 (2 m, 1f)	-	-	-
*Hyalomma scupense*	El Ghorra, El Tarf.	Cattle (123)	94 (41 m, 53f)	20 (10 m, 10f)	6/20 (2 m, 4f) *Ri. aeschlimannii*	-	-
		Sheep (250)	1 (1f)	1 (1f)	-	-	-
*Hyalomma anatolicum excavatum*	El Ghorra, El Tarf.	Cattle (123)	105 (54 m, 51f)	20 (10 m, 10f)	6/20 (4 m, 2f) *Ri. aeschlimannii*	-	-
*Hyalomma marginatum*	El Ghorra, El Tarf.	Goats (128)	4 (4f)	4 (4f)	-	-	-
*Ixodes ricinus*	El Ghorra, El Tarf.	Sheep (250)	1 (1 m)	1 (1 m)	-	-	-
	Cheabat El Balout, Souk Ahras	Mongoose (1)	1 (1 m)	1 (1 m)	-	-	-
*Ixodes hexagonus*	El Ghorra, El Tarf.	Dogs (50)	2 (2f)	2 (2f)	-	-	-
	Cheabat El Balout, Souk Ahras	Hedgehogs (4)	9 (1 m, 8f)	9 (1 m, 8f)	-	-	-
*Dermacentor marginatus*	Cheabat El Balout, Souk Ahras	Boars (2)	3 (3f)	3 (3f)	2/3 (2f) *Ri. slovaca*	-	-
*Haemaphysalis punctata*	Cheabat El Balout, Souk Ahras	Boars (2)	1 (1f)	1 (1f)	1/1 (1f) *Ri. slovaca*	-	-
*Ixodes vespertilionis*	Cheabat El Balout, Souk Ahras	Bats (10)	19 (2f, 17 nymphs)	19 (2f, 17 nymphs)	-	12/19 (1f, 11 nymphs) *B. tamiae*	3/19 (2f, 1 nymph)
*Ischnopsyllus intermedius*	Cheabat El Balout, Souk Ahras	Bats (10)	3 (3f)	3 (3f)	-	-	-
*Ctencephalides felis*	Cheabat El Balout, Souk Ahras	Hedgehogs (4)	2 (2f)	2 (2f)	2/2 (2f) *Ri. felis*	-	-
*Pariodontis riggenbachi*	Cheabat El Balout, Souk Ahras	Porcupine (1)	21 (3 m, 18f)	21 (3 m, 18f)	-	-	-
*Nycteribiidae*	Cheabat El Balout, Souk Ahras	Bats (10)	11(5 m, 6f)	11(5 m, 6f)	-	8/11 *B. tamiae*	-
*Archaeopsylla erinacei*	Cheabat El Balout, Souk Ahras	Hedgehogs (4)	110 (39 m, 71f)	110 (39 m, 71f)	80/110 (19 m, 61f) *Ri. felis*	-	-

For the investigation on wild animals and their ectoparasites in Cheabat El Balout, 77 ticks, 136 fleas, 11 *Nycteribiidae* (Table 2) and 16 spleens were sampled (two spleens from boars, one from a mongoose, two from jackals, one from a porcupine and 10 from bats).

### Detection of *Bartonella* spp.

All the selected 191 ticks of El Ghorra (Table 2) tested negative for *Bartonella* spp. However, out of all ticks, fleas, *Nycteribiidae* and spleen portions, 26 samples collected from bats were positive, including 12/19 (63.2 %) of *I. vespertilionis* ticks, 8/11 (72.7 %) *Nycteribiidae* flies and 6/10 (60 %) spleens. The results showed that all sequences of *Bartonella* spp. detected in ectoparasites and bat spleens were similar to the sequence of *B. tamiae* (100 % similarity with the *Bartonella tamiae* strain Th339 16S-23S ribosomal RNA intergenic spacer, partial sequence, GenBank no EF605284.1, 451/451 bp).

### Detection of *Rickettsia* spp.

In El Ghorra, 29/191 (15.2 %) of ticks tested positive for *Rickettsia* spp. by qPCR. These included 15/29 (51.7 %) *R. sanguineus* sensu lato, 2/29 (6.9 %) *R. bursa,* 6/29 (20.7 %) *Hy. scupense* and 6/29 (20.7 %) *Hy. a. excavatum*. Concerning the 15 *R. sanguineus* sensu lato that were *Rickettsia* spp.-positive, 3 were collected from cattle, 11 from sheep and 1 from dogs. The two *R. bursa* were sampled from cattleas well as all six *Hy. scupense*. Finally, the 6 *Hy. a. excavatum* were sampled from goats. DNA sequence analyses of the PCR products targeting *OmpA* on the *R. sanguineus* sensu lato and *R. bursa* ticks showed 100 % similarity with *Rickettsia massiliae* (GenBank accession no. U43793.1), regardless of the host’s tick type. In addition, the sequencing of the *OmpA* gene fragment from the positive *Hy. scupense* and *Hy. a. excavatum* showed 100 % similarity with *Rickettsia aeschlimannii* strain EgyRickHimp-El-Arish-17 outer membrane protein A (*OmpA*) gene, partial cds (GenBank accession no. HQ335158.1, 633/633 bp).

In Cheabat el Balout, the RKND03 qPCR system was used to test the 77 ticks, 136 fleas, 11 *Nycteribiidae* flies and 16 spleens sampled from wild animals. Overall, we detected DNA from *Rickettsia* spp. in three ticks from boars (two *D. marginatus* and one *Hae. punctata*), in 80 *A. erinacei* and two *C. felis* fleas from hedgehogs. The DNA sequence of the two rickettsia-positive *D. marginatus* and *Hae. punctata* showed 100 % similarity with *Rickettsia slovaca* strain WB2/Dm Pavullo outer membrane protein A (*OmpA*) gene, partial cds GenBank accession no. HM161787.1, 633/633 bp). Concerning the 80 *A. erinacei* and 2 *C. felis* of hedgehogs positive for *Rickettsia* spp, all 82 fleas were positive for *Ri. felis* (Table 2).

### Detection of *Coxiella burnetii*

In El Ghorra, all the 191 screened ticks were negative, however in Cheabat el Balout, we detected DNA from *C. burnetii* in three *I. vespertilionis* of bats. We confirmed the result using the second qPCR system (Table 2).

### Detection of *Borrelia* spp.

All tested samples were negative for *Borrelia* spp. using the 16S qPCR system from the *Borrelia* genus.

## Discussion

This investigation reports the first direct evidence of DNA from *B. tamiae* in *I. vespertilionis*, *Nycteribiidae* and bat spleens in Algeria. The association between the DNA from *Ri. slovaca* and *Hae. punctata* from boars and also between *Ri. massiliae* and *R. bursa* from cattle are reported for the first time in Algeria. Other rickettsiae were detected in this field as previously detected in Algeria, namely *Ri. massiliae* in *R. sanguineus* sensu lato, *Ri. aeschlimannii* in *Hyalomma* spp. ticks and *Ri. felis* in *A. erinacei* and in *C. felis* fleas. Using molecular tools *C. burnetii*, the agent of Q fever, was also detected in *I. vespertilionis* ticks of bat. Since ticks were removed from animals that in some cases could have been bacteremic, the ticks can be vectors for some pathogens but also only carriers in other cases. As a consequence, we cannot consider the presence of bacteria in the ectoparasite as proof of vector competence.

Bartonella-associated illnesses occur worldwide, and they encompass a broad clinical spectrum, including fever, skin lesions, endocarditis, lymphadenopathy, and abnormalities of the central nervous system, eye, liver and bone tissues [37]. *Bartonella tamiae* is a newly described bacterial species, initially isolated from the blood of three hospitalized patients in Thailand [33]. These patients presented with headache, myalgia, anemia, and mild liver function abnormalities [38]. This novel *Bartonella* species has been newly recognized as a pathogen [33, 39]. Throughout our investigation, *B. tamiae* was detected for the first time in Algeria, and in ticks, *Nycteribiidae* and bat spleens. Bats are the second species group of mammals after rodents confirmed to carry *Bartonella* spp. [40]. Renewed interest in *Bartonella* research in mammals has confirmed the presence of *Bartonella* spp. in bats in Guatemala, Kenya [41] and the United Kingdom [42].

*C. burnetii* was also detected in this study; this pathogenic agent of Q fever is associated with many manifestations [26, 43]. Q fever is typically an acute febrile illness with nonspecific clinical signs in humans, but isolated fever, hepatitis and/or atypical pneumonia are the most commonly described manifestations. A small proportion of infected people develop life-threatening valvular endocarditis [26, 43]. Q fever has been described worldwide in outbreaks involving sheep, goats, cats, dogs and wild animals, while reservoirs are extensive but only partially known and include mammals, birds, and arthropods, mainly ticks [44]. In Algeria, few human cases have been reported, including one in 2005, where two patients were found to be seropositive for *C. burnetii* (one was confirmed positive by nested PCR) [45]. In 2012, through 268 qPCR-tested samples from Oran, Western Algeria, only one patient was positive for *C. burnetii* [27].

*B. tamiae* and *C. burnetii* were detected in *I. vespertilionis*. This tick species parasitizes bats specifically [46, 47]. Humans can also act as accidental hosts [48]. *I. vespertilionis* represents little interest for human health because it is restricted to the darkest part of bat caves [49]. In literature, no evidence was found to prove that this species can transmit pathogens to humans or other animals [49]. We detected *Ri. slovaca* in ticks from boars, namely in *Hae. punctata* and *D. marginatus*. Our detection of *Ri. slovaca* in *Hae. punctata* ticks may be due to co-feeding with infected *D. marginatus*, which is a recognized vector and reservoir of the bacteria. Literature reported also the possibility of transmission to other tick species by feeding on bacteremic animals, as is the case of *Ri. slovaca* [50]. *Ri. slovaca* is associated with a syndrome characterized by scalp eschar and neck lymphadenopathy following tick bites [51]. In Algeria, *Ri. slovaca* was previously detected in *D. marginatus* ticks collected from vegetation in the Blida region, in 2012 [52].

In Algeria, *Ri. massiliae* was detected in *R. turanicus,* in 2006 [53]. Our results confirm the presence of *Ri. massiliae* in Algerian ticks, where we detected it in *R. sanguineus* sensu lato from cattle, sheep, dogs and boars. Using qPCR, it was also detected in *R. bursa* from cattle. These SFG rickettsiae were described in 1992, and then subsequently detected in other *Rhipicephalus* spp., including *R. bursa* in European countries [51].

Our results indicate the presence of *Ri. aeschlimannii* in *Hy. a. excavatum* and *Hy. scupense* ticks from cattle in the far northeast of Algeria. *Ri. aeschlimannii* is an emerging pathogen that causes symptoms similar to those of Mediterranean spotted fever [51]. It has been associated with ticks, particularly with *Hy. marginatum* and *Hy. rufipes* ticks, in southern Europe and Africa [51].

In Algeria, *Ri. aeschlimannii* was previously detected in *Hy. dromedarii* and *Hy. rufipes* from camels from southern Algeria [54] and *Hy. aegyptium* ticks from tortoises trapped near Algiers [55]. Our results confirm the presence of *Ri. aeschlimannii* in Algeria but also complete the geographical distribution of this pathogen from the south and center to the far northeast.

Finally, we detected DNA from *Ri. felis* in hedgehog fleas (*Ct. felis and A. erinacei*). *Ri. felis* is an emergent agent of infectious diseases in humans, and this SFG agent is known to be maintained in cat fleas (*Ct. felis*) [56, 57]. To date, 12 flea species, eight tick species and three mite species have been found to be infected with *Ri. felis* [57]. These rickettsiae have also recently been detected in several mosquito species in sub-Saharan Africa [31, 58, 59]. Clinical features may include fever, fatigue, headache, generalized maculopapular rash and inoculation eschar(s) [57]. It is known to be a frequent agent of fever of unknown origin [60]. In Algeria *Ri. felis* was previously detected in *A. erinacei* fleas of hedgehogs of M’sila and Bordj-Bou-Arreridj, Algeria [61, 62]. It was also detected in *Ct. canis* [60]. Here, we report for the first time the presence of *Ri. felis* in *Ct. felis* fleas from Algeria.

## Conclusion

For the first time in Algeria, we detected *B. tamiae*, *Coxiella burnetii* and rickettsiae (*Ri. slovaca, Ri. massiliae, Ri. aeschlimannii* and *Ri. felis*) in two regions of the far northeast of Algeria. We expanded knowledge of the repertoire of ticks and flea-borne bacteria present in ectoparasites and/or tissues of domestic and wild animals in Algeria. Our findings will help human and veterinary clinicians to enlarge the spectrum of pathogens to consider in differential diagnosis. Future studies on rickettsioses, bartonelloses and other vector-borne diseases should be performed to assess their epidemiological and clinical relevance in Algeria, to estimate the actual prevalence and to allow the establishment of anti-vector control plans.
